# Novel Metrics for High-Density sEMG Analysis in the Time–Space Domain During Sustained Isometric Contractions

**DOI:** 10.1109/OJEMB.2024.3449548

**Published:** 2024-08-26

**Authors:** Giovanni Corvini, Michail Arvanitidis, Deborah Falla, Silvia Conforto

**Affiliations:** Department of Industrial, Electronic and Mechanical EngineeringUniversity of Roma Tre 416776 00154 Rome Italy; School of Sport, Exercise and Rehabilitation Sciences, College of Life and Environmental SciencesUniversity of Birmingham 1724 Birmingham B15 2TT U.K.

**Keywords:** HD-sEMG, muscle fatigue, spatial muscle distribution, spatiotemporal analysis, endurance time

## Abstract

*Goal:* This study introduces a novel approach to examine the temporal-spatial information derived from High-Density surface Electromyography (HD-sEMG). By integrating and adapting postural control parameters into a framework for the analysis of myoelectrical activity, new metrics to evaluate muscle fatigue progression were proposed, investigating their ability to predict endurance time. *Methods:* Nine subjects performed a fatiguing isometric contraction of the lumbar erector spinae. Topographical amplitude maps were generated from two HD-sEMG grids. Once identified the coordinates of the muscle activity, novel metrics for quantifying the muscle spatial distribution over time were calculated. *Results:* Spatial metrics showed significant differences from beginning to end of the contraction, highlighting their ability of characterizing the neuromuscular adaptations in presence of fatigue. Additionally, linear regression models revealed strong correlations between these spatial metrics and endurance time. *Conclusions:* These innovative metrics can characterize the spatial distribution of muscle activity and predict the time of task failure.

## I. Introduction

Muscle fatigue is defined as a transient decrease in the capacity of generating muscle force [1]. Based on the level of the motor pathway where this event occurs, fatigue can be distinguished between central fatigue and peripheral fatigue [2]. The myoelectrical signals recorded on the surface of the skin, directly reflect the underlying biochemical and physiological changes occurring within muscles, making surface electromyography (sEMG) a preferred tool usually applied to study muscle fatigue [3]. Many techniques have been developed to extract sEMG parameters able to assess the onset and progression of muscle fatigue [4]. Most research has primarily focused on studying changes in either the temporal or spectral domain [5], as well as studying the combined analysis of EMG joint analysis of spectrum and amplitude (JASA) features [6] to discriminate fatigue-induced changes. Nevertheless, these studies did not consider the possibility that changes in sEMG parameters could occur due to the spatial redistribution of muscle activity [7]. To explore these neuromuscular adaptations, High-Density surface EMG (HD-sEMG) can be applied; this technique involves the placement of multiple electrodes on a single muscle or muscle region to identify and quantify the spatial distribution of muscle activity through time [8]. This is typically achieved by calculating either the Centre of Gravity (CoG), which is the centroid of the sEMG amplitude distribution, or the weighted centroid Region of Activation (RoA) of the muscle, usually characterized by a muscular activity greater than 70% of MVC, as described in [9]. Redistribution of muscle activity is believed to have physiological importance because it could help recruiting new muscle fibers [10], and thus minimize fatigue by redistributing the load between muscle regions [11]. While the spatial distribution was found to be task-dependent [12] in a pre-fatigue stage, similar muscle strategies were observed in a later stage of muscle fatigue for maintaining performance [13] [14], with highly reliable patterns of sEMG activity [15]. Spatial redistribution could prolong endurance by preventing an excessive strain on the muscle fibers engaged at the beginning of the task [16]. A previous study showed a progressive redistribution of upper trapezius activity during sustained shoulder abduction and found an association between the amount of shift of the centroid of the sEMG amplitude map and the endurance time in healthy volunteers [17]. A similar observation was found for the lumbar Erector Spinae (ES) while performing a fatiguing contraction (i.e., a sustained lumbar extension) [18]. Likewise, the spatial distribution of lumbar ES activity was also investigated during a trunk extension endurance task, showing that a larger redistribution was associated with a longer period of contraction [19]. Collectively, these studies showed variability in the extent of muscle activity redistribution between individuals. However, each study examined changes in the position of the x- and y- coordinates of the centroid of activity, and to the best of our knowledge, no specific metrics have been defined to quantify and characterize the movement of the activity centroid in either domain, i.e., time and space. By doing so, new metrics may reveal even greater associations between sEMG variables and endurance time, offering the possibility of even being able to predict the onset of muscle fatigue. This would have implications in many fields including rehabilitation and sport science. Drawing inspiration from measures of postural control and stability, we considered parameters usually extracted from the Centre of Pressure (CoP) [20], [21] to define new metrics for analyzing spatial aspects of HD-sEMG signals. The rationale lies in the fact that by quantifying the shift of the CoP, we gain insights into how the body maintains balance by adjusting load distribution. Similarly, by tracking and quantifying muscle activity within the muscle itself, we could understand how the neuromuscular system adapts to fatigue by recruiting new fibers across different muscle regions, thereby redistributing myoelectrical activity spatially. We hypothesized that these novel spatial features might reveal greater associations with the endurance time. This approach may enhance the ability to predict the onset of muscle fatigue, with significant implications for rehabilitation and sports science.

## II. Materials and Methods

**Figure 1.**
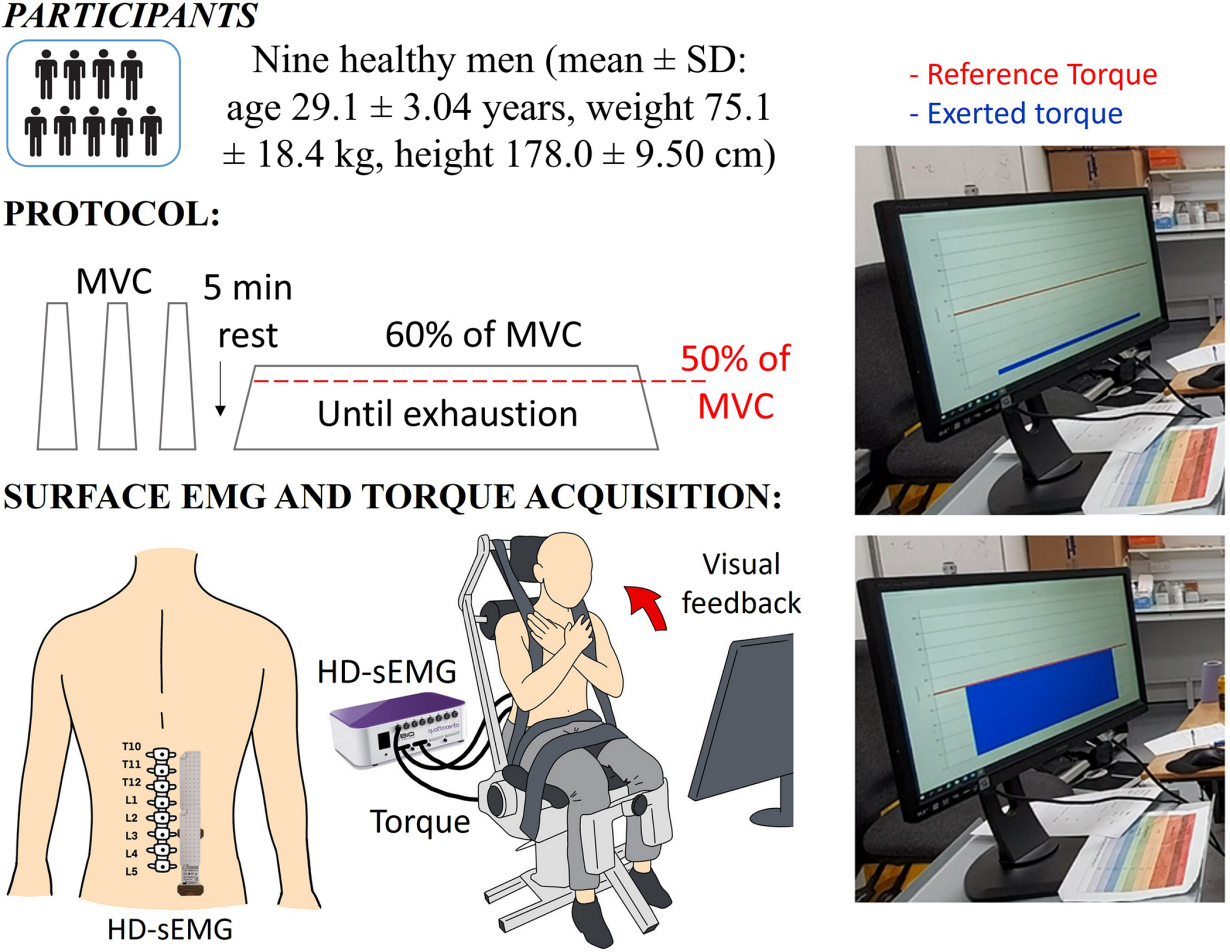
Top left: Participants and their anthropometric characteristics. Middle left: Fatiguing protocol. Bottom left: Positioning of two HD-sEMG grids on the Erector Spinae and representation of a participant sat on the Biodex performing back extension. Right: Visual feedback provided during the fatiguing contraction on a screen placed in front of the participants.

The design of the study, the description of the protocol and the characteristics of the participants, summarized in Fig. 1, are fully described in the supplementary materials.

Participants were asked to perform a fatiguing contraction at 60% of the peak value of the Maximum Voluntary Torque (MVT). Visual feedback, together with verbal encouragement, was provided during the entire task. Task failure was reached when the exerted torque level of the participant dropped to 50% of MVT or less (i.e., more than 10%) for a minimum of three consecutive seconds.

### A. Surface EMG and Torque Acquisition

HD-sEMG signals were detected from the right lumbar Erector Spinae while performing a back extension, as described in [22], with two semi-disposable adhesive grids of 64 electrodes each (13x5 electrodes, without one electrode in the upper corner to define the grid orientation). The isokinetic dynamometer, with chair attachment, was used to record the torque generated during the back extension. Visual feedback was given to the participant showing both the reference torque level to be reached (represented by a constant red line indicating the required level of force to be exerted), and the exerted torque (represented by a dynamic blue bar, changing according to the participant's muscle output), as shown in Fig. 1.

### B. Data Processing

**Figure 2.**
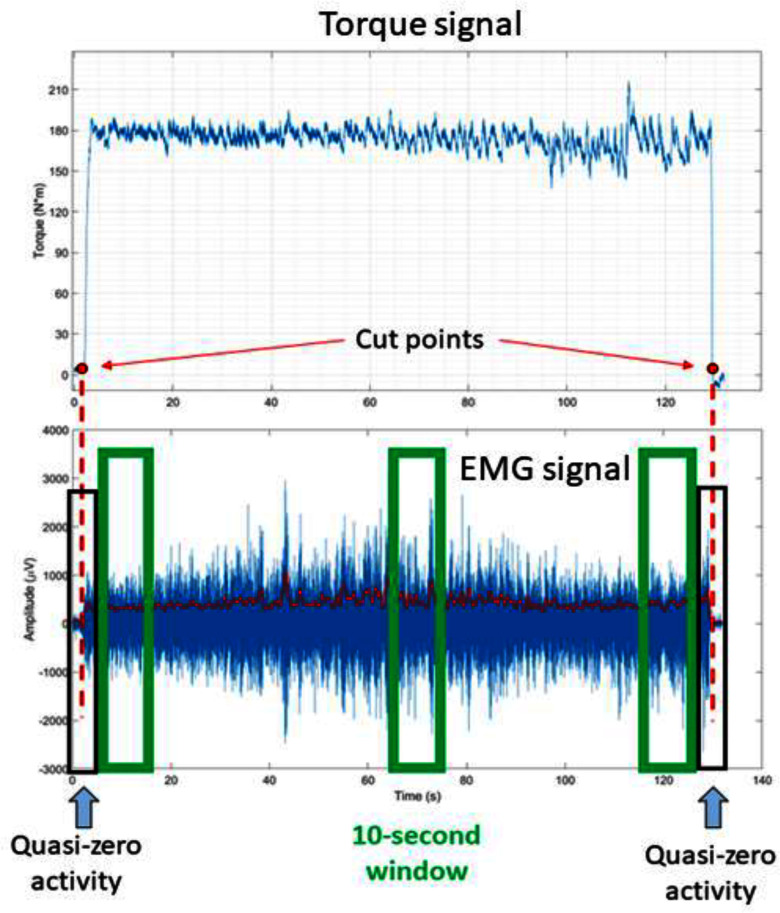
Torque signal acquired from the Biodex during a back extension contraction. Once identified the transient phases (cut points), two 4-second windows were identified (“quasi-zero activity”). Signal processing was applied on signals within these two “quasi-zero activity” windows. Statistical analysis was performed on metrics extracted from the three segments identifying beginning (T 1), middle (T 2) and end (T 3) of the contraction.

Data was processed and analyzed offline in MATLAB (The MathWorks Inc., Natick, Massachusetts). The torque signal was filtered with a low pass filter (3rd order Butterworth) with a 10 Hz cut-off frequency and was used to segment the data. First, the transient phases of the torque level, specifically the ascending and descending segments from 0% to 60% and vice versa, were manually marked. Subsequently, two 4-second windows spanning two seconds before and two seconds after the marked points were defined. These time intervals, which were called “quasi-zero activity” segments, were used as boundaries for our analysis. Processing was applied on the portion of signals enclosed between the two “quasi-zero activity” segments, as shown in Fig. 2.

The two HD-sEMG grids were processed together after being considered as one single larger matrix (26x5). Monopolar HD-sEMG signals were manually inspected, and those with a low Signal-to-Noise Ratio (SNR), marked as bad channels, were removed. Signals were digitally filtered (3rd order Butterworth filter) with a bandpass filter in the bandwidth 20-450 Hz and a notch at 50 Hz to remove the Power Line noise. To characterize the amplitude of the signals, the Root Mean Square (RMS) envelope was extracted by smoothing the RMS values of the signals with a moving average method using a window length equal to 500 milliseconds. For the frequency analysis, the spectrogram was estimated with a time resolution of 500 milliseconds with no overlap, and the Average of Instantaneous Frequency (AIF) was computed. Although we assumed that the signals were pseudo-stationary in this time interval, we used the AIF because it is inherently suitable for nonstationary signals [23].

**Figure 3.**
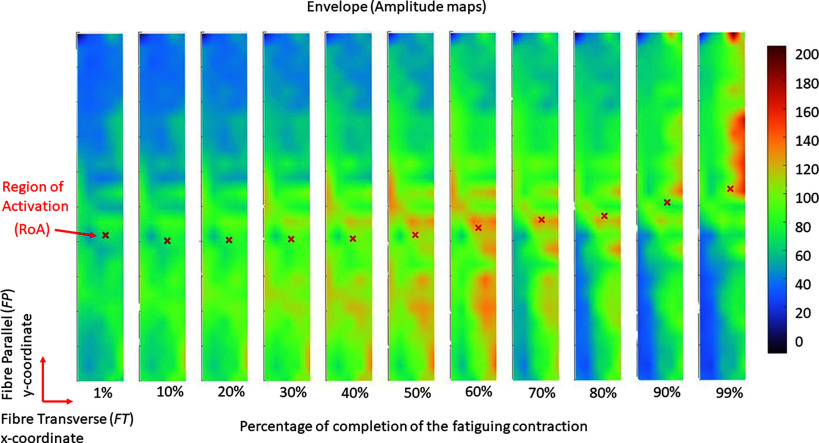
Example of topographical maps extracted from monopolar HD-sEMG signals. All maps were calculated in the portion of signals enclosed between the two “quasi-zero activity” segments. Red crosses represent the Region of Activation (RoA), which was defined as the weighted centroid of the electrical activity greater than 70% of the local maximum. For visualization only, bad channels were replaced by the interpolation of adjacent channels.

The 128 envelopes extracted from monopolar signals were used to create topographical maps of muscle activity, as shown in Fig. 3. Bad channels were excluded during the extraction of the parameters and the analysis. The x- and y-coordinates, which we defined as Fiber-Transverse (*FT*) and Fiber-Parallel (*FP*) directions, respectively, were identified. From each of these topographical maps, once identified the *FT* and *FP* directions, we calculated the Region of Activation. The RoA was calculated as the weighted centroid of the electrical activity greater than 70% of the local maximum (maximum RMS amplitude), which was calculated at each time instant, obtaining the x- and y-coordinates ($\text{RoA}_{FT}^0[n],\,\text{RoA}_{FP}^0[n]$) evolving over time. More details are described in the supplementary material.

Using the formula from Prieto et al. [20], several metrics, which are typically calculated from the CoP to study postural control, have been derived and adapted. The mathematical derivation of these metrics is provided in the supplementary material. Once computed, each metric has been averaged over three different 10-second segments (S) at the beginning (T 1), in the middle (T 2), and at the end (T 3) of the contraction, as shown in Fig. 2.

### C. Statistical Analysis

The statistical analysis was performed on the metrics derived from the RoA, assuming that similar results would be obtained if considering the Centre of Gravity (CoG), which is defined as the weighted centroid of the HD-sEMG grid. This assumption was made because of the positive strong correlation between the two measures found in the work of Falla et al. [16].

First, we tested the normality of the data using the Shapiro-Wilk test. For data following a non-normal distribution, the non-parametric Friedman test for repeated measures was used to assess the differences of the variables at different time intervals (i.e., beginning, middle, and end of the contraction). The Kendall's coefficient was used as measure of the Friedman test effect size, using Cohen's interpretation guidelines: small effect (0 - 0.299), moderate effect (0.3 - 0.499), and large effect (0.5 – 1). A significant Friedman test was followed up by pairwise Wilcoxon signed-rank tests for identifying which time instants were different. In the following we will refer to T 1, T 2, and T 3 to the different time window at the beginning (T 1), in the middle (T 2) and at the end of the contraction (T 3). Bonferroni correction was used to correct for the multiple comparisons. The significance levels were set to alpha equal to 0.05 and 0.01. The significance level is identified by the asterisks: * p<0.05 and ** p<0.01. Finally, for the prediction of the time endurance, we conducted a linear regression analysis to explore the association between the provided metrics and the time of exhaustion, using the R^2^ value and the corresponding p-value with alpha equal to 0.05.

## III. Results

Since most of the analyzed data did not follow a normal distribution, as evidenced by the significant results of the Shapiro-Wilk test (p<0.05), we applied the non-parametric Friedman test for repeated measures. All results of the statistical analysis are presented in Table I.

**Figure 4.**
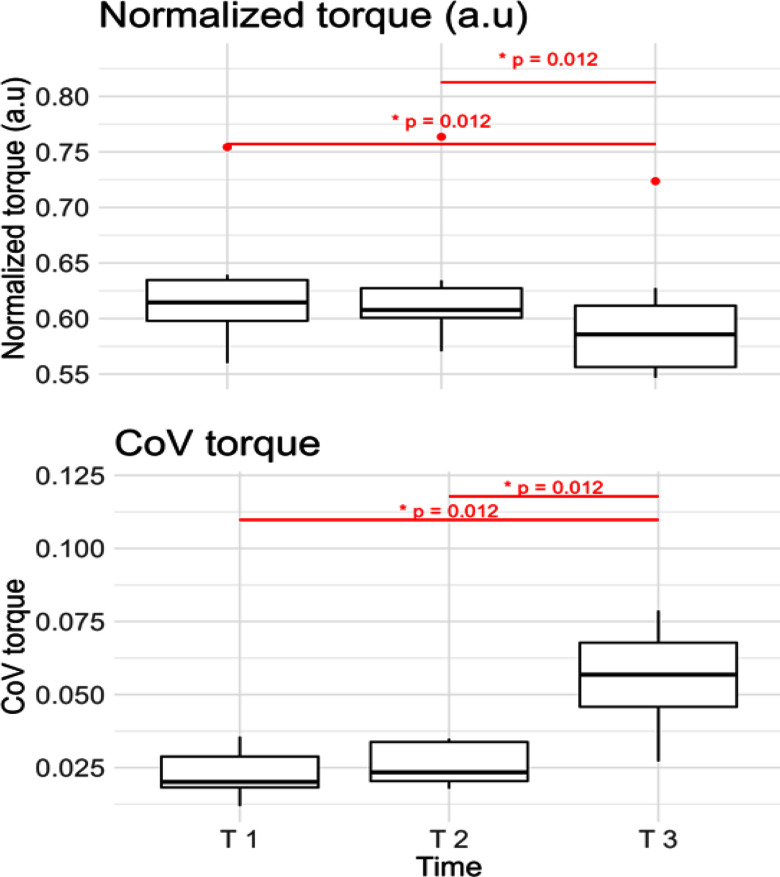
Upper plot: The normalized level of torque computed at the beginning (T 1), at the midpoint (T 2) and at the end (T 3) of the contraction. Lower plot: Coefficient of Variation of the normalized torque (range 0-1).

In Fig. 4 it is possible to observe a decrease of the exerted torque by about 5% from the beginning to the end of the contraction, whereas the Coefficient of Variation (CoV) of torque steadily rises. The decrease of torque and the increase in its variability were statistically significant from T1 to T 3 and from T 2 to T 3, but not from T 1 to T 2.

**Figure 5.**
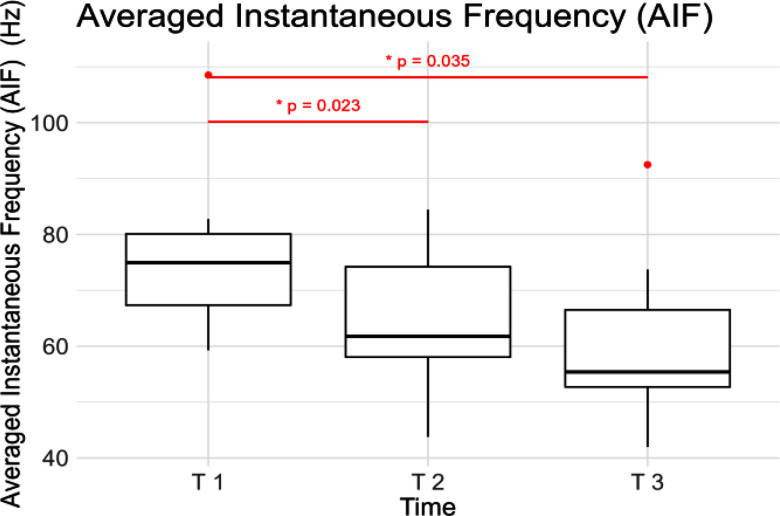
Averaged Instantaneous Frequency (AIF) computed at the beginning (T 1), at the midpoint (T 2) and at the end of the contraction (T 3).

When analyzing the amplitude and the spectrum of sEMG signals, different behaviors over time were detected. The increase of the sEMG envelope over time is not statistically significant. On the contrary, for the AIF, as shown in Fig. 5, a significant decrease (p<0.05) of approximately 15 Hz was found from the beginning to the midpoint, and a decrease of approximately 25 Hz was observed from the beginning to the end of the contraction (p<0.05).

**Figure 6.**
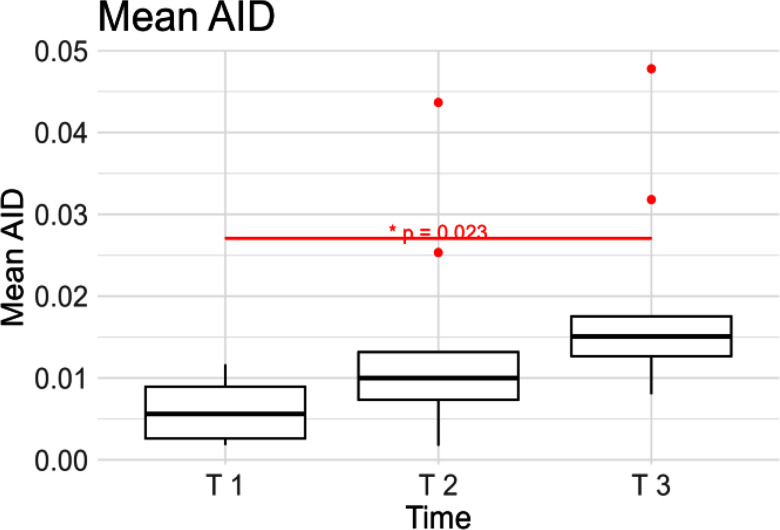
Mean Activation Intensity Distance (AID) computed at the beginning (T 1), at the midpoint (T 2) and at the end of the contraction (T 3).

In Fig. 6, a significant increase (p<0.05) was observed in the Mean Activation Intensity Distance (AID) from T 1 to T 3, while from T 1 to T 2 and from T 2 to T 3, the increase was not significant (p>0.05)*.* Since this measure is derived from the Mean AID calculated separately for the two different coordinates (AID*_FT_* and AID*_FP_*), a similar increasing trend was observed for each metric singularly. However, when analyzing the *FP* direction, we noticed significant change from T 1 to T 3 (p<0.05), while on the *FT*, the increase was found to significant (p <0.05) both from T 1 to T 2 and from T 1 to T 3, as can be seen in Table I.

**Figure 7.**
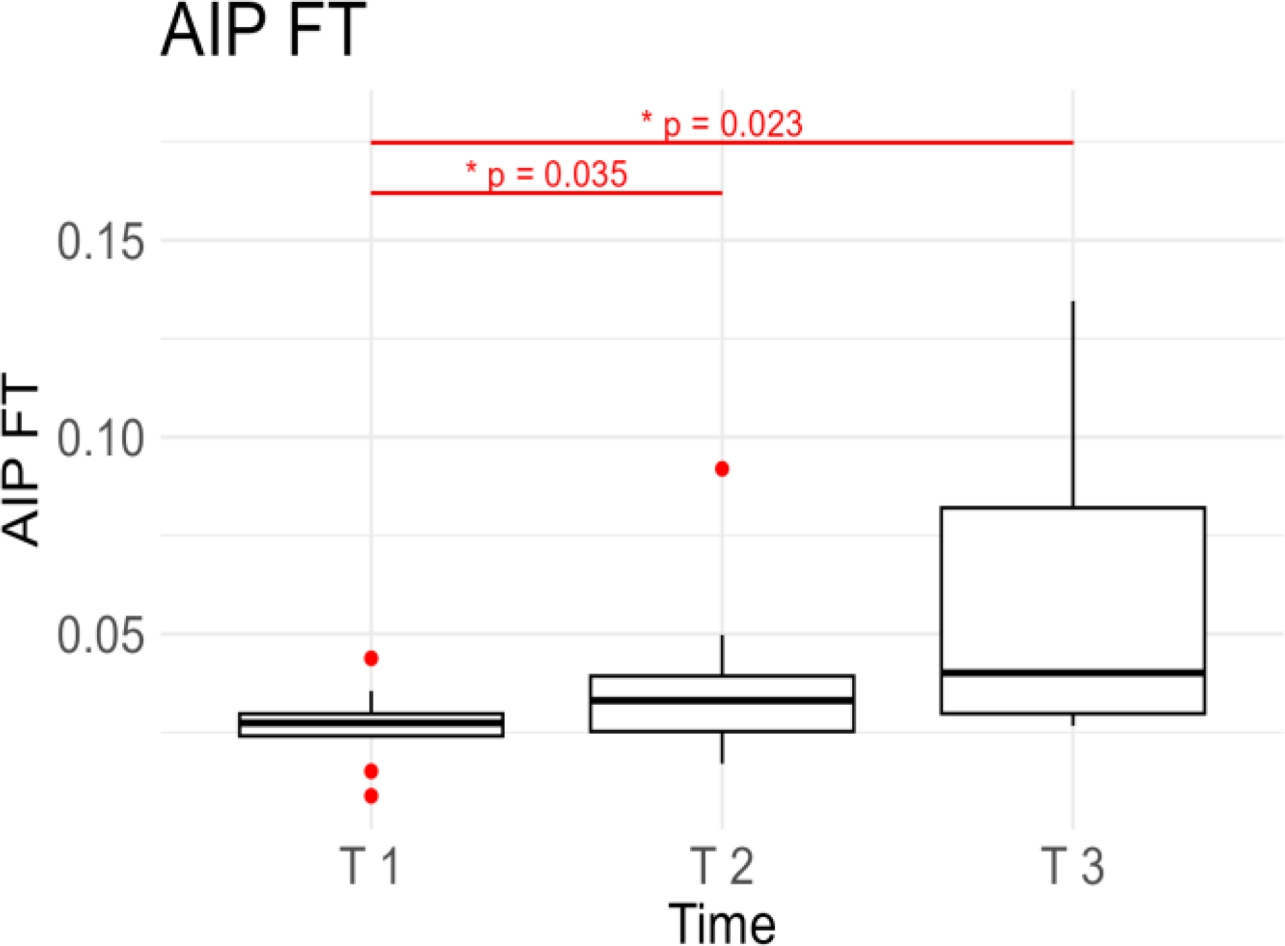
Activation Intensity Path on Fiber Transverse direction (AIP*_FT_*) computed at the beginning (T 1), at the midpoint (T 2) and at the end of the contraction (T 3).

In contrast, when analyzing the Total AIP, as well as the Mean AIV that is obtained by dividing the AIP with respect to the time window, no significant differences were found across the three different time intervals. Nonetheless, when we analyzed the individual contributions in the two directions, distinct patterns emerged. Specifically, the total excursion on the *FP* direction is an order of magnitude larger than the one produced on the *FT* direction. Upon individual analysis, we observed that while there were no significant changes in Total AIP*_FP_*, a significant increase (p<0.01) was evident in the *FT* direction (Total AIP*_FT_*), with significant differences between T 1 and T 3, as well as between T 2 and T 3. The latter are shown in Fig. 7. The results for the Mean AIV mirrored those of the Total AIP because these two metrics are directly proportional to each other, as well as the metric computed in the individual directions.

**TABLE 1:** All Results of the Statistical Analyses Are Summarized. Friedman Test for Repeated Measures (N = 9) Was Used Because of the Non-Normal Distribution of the Data. When the Test Was Significant, a Wilcoxon Post-hoc Test With Bonferroni Correction Was Performed to Explore the Pairwise Comparisons Among the Time Interval (Beginning T 1, Middle T 2, and End of Contraction T 3). Kendall's Coefficient Was Used as Measure of the Friedman Test Effect Size. Significance Level Is Identified as * p<0.05 *and* ** p<0.01

**Variable**	Effect size	Statistic	Friedman P-value	Wilcoxon test pairwise comparison	Adjusted p-value
**Torque**	Large	13.55	** 0.0011	T 1 – T 2	1
				T 1 – T 3	* 0.012
				T 2 – T 3	* 0.012
**CoV of torque**	Large	14	** 0.0009	T 1 – T 2	0.903
				T 1 – T 3	* 0.012
				T 2 – T 3	* 0.012
**Envelope**	Moderate	8.22	* 0.016	T 1 – T 2	0.08
				T 1 – T 3	0.29
				T 2 – T 3	1.000
**Averaged Instantaneous Frequency (AIF)**	large	9.55	** 0.0084	T 1 – T 2	* 0.023
				T 1 – T 3	* 0.035
				T 2 – T 3	0.750
**Mean Activation Intensity Distance (AID)**	Moderate	8	* 0.01831	T 1 – T 2	0.117
				T 1 – T 3	* 0.023
				T 2 – T 3	0.387
**Mean AID on Fiber Parallel direction (AID*_FP_*)**	Moderate	8	** 0.01831	T 1 – T 2	0.117
				T 1 – T 3	* 0.023
				T 2 – T 3	0.387
**Mean AID on Fiber Transverse direction (AID*_FT_*)**	Large	14.88	** 0.0005	T 1 – T 2	* 0.012
				T 1 – T 3	* 0.012
				T 2 – T 3	0.223
**Mean Activation Intensity Velocity (AIV)**	Small	1.55	0.4594	—	—
**Mean AIV on Fiber Parallel direction (AIV*_FP_*)**	Small	0.88	0.6411	—	—
**Mean AIV on Fiber Transverse direction (AIV*_FT_*)**	Large	9.55	** 0.0084	T 1 – T 2	* 0.035
				T 1 – T 3	* 0.023
				T 2 – T 3	0.387
**Activation Ellipse Zone (AEZ)**	Large	12.66	** 0.0017	T 1 – T 2	* 0.023
				T 1 – T 3	* 0.012
				T 2 – T 3	0.117
**Activation Movement Zone (AMZ)**	Large	10.88	** 0.0043	T 1 – T 2	* 0.012
				T 1 – T 3	* 0.023
				T 2 – T 3	0.903
**Activation Circle Zone (ACZ)**	Moderate	6.88	* 0.0319	T 1 – T 2	0.117
				T 1 – T 3	* 0.023
				T 2 – T 3	0.387
**Activation Intensity Spread (AIS)**	Moderate	8	* 0.0183	T 1 – T 2	0.164
				T 1 – T 3	* 0.023
				T 2 – T 3	0.293
**Standard deviation on Fiber Transverse (std*_FT_* coordinate)**	Large	14.88	** 0.0005	T 1 – T 2	* 0.012
				T 1 – T 3	* 0.012
				T 2 – T 3	0.223
**Standard deviation on Fiber Parallel (std*_FP_* coordinate)**	Moderate	8	* 0.0183	T 1 – T 2	0.164
				T 1 – T 3	* 0.023
				T 2 – T 3	0.293
**Average on Fiber Transverse (avg*_FT_* coordinate)**	Small	2.88	0.2358	—	—
**Average on Fiber Parallel (avg*_FP_* coordinate)**	Small	1.55	0.4594	—	—

**TABLE 2:** Linear Regression Between Endurance Time and All the Variables. We Reported R^2^ and the P-Value Associated to the Model. Values Exceeding 0.5 Are Highlighted in Green, While the Significance of P-Values Was Indicated by Asterisks

Variable	R^2^	P-value
Max torque	0.356	0.0897
Max CoV torque	0.188	0.2431
Max Envelope	0.0001	0.9581
Max AIF	0.126	0.3483
Displacement	0.505	* 0.0318
Max Mean AID	0.460	* 0.0444
Max Mean AID*_FP_*	0.438	0.0519
Max Mean AID*_FT_*	0.894	** 0.0001
Max Mean AIP	0.102	0.4008
Max Mean AIP*_FP_*	0.131	0.3373
Max Mean AIP*_FT_*	0.779	** 0.0016
Max Mean AIV	0.230	0.1911
Max Mean AIV*_FP_*	0.260	0.1603
Max Mean AIV*_FT_*	0.688	** 0.0056
Max AEZ	0.828	** 0.0006
Max AMZ	0.206	0.2187
Max ACZ	0.545	* 0.0231
Max AIS	0.533	* 0.0255
Max avg *FP*	0.156	0.2913
Max avg *FT*	0.884	** 0.0001
Max std *FP*	0.156	0.2913
Max std *FT*	0.883	** 0.0001

**Figure 8.**
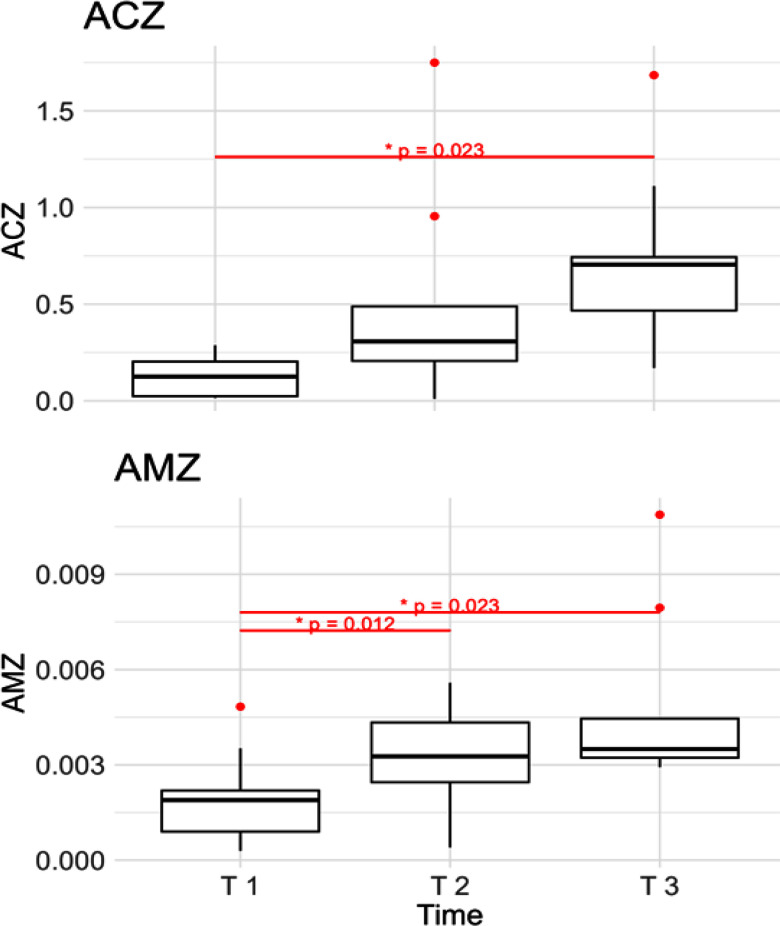
Upper plot: Activation Circle Zone (ACZ) computed at the beginning (T 1), at the midpoint (T 2) and at the end of the contraction (T 3). Lower plot: Activation Movement Zone (AMZ).

Fig. 8 depicts the ACZ and AMZ, two metrics associated with the calcuation of areas covering the full path length travelled by the RoA over time. As illustrated, both metrics reveal an upward trend towards the end of the contraction. While the AMZ presented a significant increase both from T 1 to T 2 and from T 1 to T 3, the ACZ showed a significant difference (p<0.05) only between T 1 and T 3. When analyzing the Activation Ellipse Zone (AEZ), we noticed a similar behaviour to the Activation Movement Zone (AMZ), with a significant difference between T 1 and T 2, in addition to the significant difference from the beginning to the end of the contraction (T 1 and T 3). Moreover, in the analysis of AEZ we noticed a large increase in the inter-variability among subjects in the final time instant considered (T 3).

When evaluating the behavior of the Activation Intensity Spread (AIS), it was observed that there was a significant difference between T 1 and T 3. By examining the single contribution provided by the standard deviation on each individual direction (*FT* and *FP*), we noticed a similar behaviour specifically in the *FP* direction. On the other hand, in the *FT* direction, we have a significant increase both between T 1 and T 2 and between T 1 and T 3. Similar to the Total Activation Intensity Path, the standard deviation on the *FP* direction is one order of magnitude larger than the one on the *FT* direction, so the Activation Intensity Spread mainly reflects the variability on the *FP* direction.

Finally, as can be seen in the last two rows of Table I, the average of the two coordinates in the three considered time intervals showed no significant differences over time.

Table II presents the R^2^ values and the corresponding p-value extracted by the regression linear model. All the values (except for the displacement, which was computed as in [17]) are obtained considering the regression between the endurance time and maxima of the described metrics. Similar results to those in the study of Farina et al. [17] were found, noticing that participants who showed the greatest redistribution of muscle activity (displacement of the RoA) had longer endurance times (R^2^ = 0.505, p < 0.05). On the contrary, no significant correlations were found for the maximum level of Torque and its maximum variability (i.e., the CoV of the Torque), nor in the common parameters usually analyzed from the surface EMG (i.e., the envelope and the averaged instantaneous frequency).

Considering the spatial metrics, we observed a positive correlation between endurance time and maximum Mean AID (R^2^ = 0.460, p < 0.05). A positive correlation was observed between the maximum Mean AID in the *FT* direction and the endurance time (R^2^ = 0.894, p < 0.05). When considering the Activation Intensity Path and the Activation Intensity Velocity, we observed a similar strong correlation of these metrics (R^2^ = 0.779 and R^2^ = 0.688, respectively, p < 0.05) with the endurance time. Correlations were also found for the metrics related to the area enclosing the entire shift of the RoA, especially the AEZ (R^2^ = 0.828, p < 0.01) and Activation Circle Zone (ACZ) (R^2^ = 0.545, p < 0.05). Finally, by considering the average and the standard deviations of the RoA along both directions, we noticed that while there was a very weak correlation between endurance time and the metric computed on the *FP* direction, there was a positive correlation with the maximum values extracted from the *FT* direction.

## IV. Discussion

The primary objective of this study was to investigate both spatial and temporal domains during sustained contractions of the erector spinae. For this purpose, we introduced novel metrics designed to capture spatiotemporal information, with the goal of characterizing the progression of fatigue. Furthermore, we explored the potential of these spatiotemporal metrics to predict time to task failure through correlation analysis. Our findings highlight the critical role of examining changes in the spatial distribution of the lumbar ES and their relationship to fatigue. As a result, the introduction of new metrics to capture the characteristics of these spatial muscle redistributions (i.e., distance, spread, velocity) might not only help in monitoring muscle fatigue but could also be useful in anticipating when task failure is likely to occur.

In alignment with previous findings [24], we observed a decline in the averaged instantaneous frequency parameter as the contraction progressed, confirming the effectiveness of the parameter in the general detection of the myoelectrical signs of fatigue. However, being a parameter representing the global status of the muscle, it may not be the best solution to identify the differences produced within different regions of the same muscle. When reviewing changes in the sEMG amplitude, calculated as a spatial average value over the grid, through the course of the contraction, an upward trend was detected. This increase is known to be attributed to the increased number of recruited MUs, to an increase in their firing rate or to a better synchronization in their discharge rates [5]. Interestingly, despite these observations, the difference in the amplitude of the sEMG signal from the beginning to the end of the contraction was not statistically significant. A potential reason for this lack of significance could be attributed to the effect of spatial averaging across the entire HD-sEMG grid. This averaging effect, providing the total muscle activity such as the one recorded with a single bipolar electrode, could not capture the differences in muscle activation. In fact, the activation of additional muscle areas might potentially counterbalance those regions with little or reduced activity. Consequently, through spatial averaging (or with single bipolar acquisition), we could obscure the true extent of muscle activity redistribution. In addition, the 10-second time segment used for averaging might further modulate these signals reducing the differences introduced by local muscle activation. This observation reinforces the necessity for the use of HD-sEMG, and the need for new metrics that could accurately consider and describe amplitude variations across specific areas of a muscle instead of providing a global picture of the muscle. Such metrics could provide detailed insights into how and where specific regions of a muscle become active as fatigue sets in, which could be exploited to develop targeted interventions or specific training programs, to mitigate the effects of muscle fatigue. By evaluating the RoA movements over the muscle region, and by applying new metrics to assess these variations, new insights have been obtained on the redistribution of muscle activity with fatigue. The Activation Intensity Distance displayed a consistent increase during the contraction. This indicates a significant displacement of the RoA from its initial position, likely as an adaptive response to fatigue. This difference between the beginning and the end was found to be significant across both *FP* and *FT* directions. Naturally, given the size of the grid (200x32mm), the amount of shift along the *FP* direction was larger than the shift along the *FT* direction, producing the main effect in the Mean AID. However, as shown in Table II, the AID*_FT_* was more strongly correlated with the endurance time, whereas AID*_FP_* exhibited a weaker correlation. This suggests that muscle fiber recruitment may be more uniform and consistent in the *FP* direction at the onset and during the entire contraction, while towards the exhaustion there appears to be an increased lateral shift (*FT* direction).

These changes along the *FP* direction have also been observed in previous studies [16], [18], [19]. More specifically, when healthy individuals perform sustained or repetitive tasks, different regions within a muscle become progressively more active [16], [25], especially with high loads [26]. This is thought to reflect a strategy to redistribute the load to different regions, thereby limiting localized muscle fatigue. For example, Sanderson et al. [19] demonstrated that redistribution of lumbar ES muscle activity occurs mainly along the *FP* direction in healthy individuals during isometric lumbar extension contractions, but without a clear preference in the direction of change (cranial or caudal). Similarly, Falla et al. [27] observed a comparable pattern during a lifting task, where the centroid shifted towards more caudal regions in the *FP* direction of the lumbar ES towards the end of the fatiguing task. In contrast, Arvanitidis et al. [28] did not observe any spatial changes in lumbar ES activity during an isokinetic dynamic fatiguing task in healthy individuals. As demonstrated by Abboud et al. [29], motor unit territories within the lumbar ES muscle are organized into two distinct regions. This finding supports the idea that regional changes in activation can occur within the same muscle, suggesting the possibility of selective activation of different muscle regions during various functional tasks. This might indicate the recruitment of newer, less-fatigued motor units within the same muscle when changes along the *FP* direction are observed.

Based on the above it is evident that most previous studies have observed changes spatial shifts in muscle activity along the *FP* direction. However, our results suggest that muscle redistribution can be seen also along the *FT* direction. These findings align with those of Marineau-Bélanger [14], who recently observed a lateral shift in lumbar ES activity near the end of a fatiguing task. This lateral shift likely indicates the engagement of adjacent muscles as a compensatory strategy to maintain motor performance. For example, a more medial pattern of activation (i.e., closer to the spine) suggests a higher contribution from the superficial multifidus at the beginning of the tasks. Conversely, a more lateral shift in muscle activity (i.e., far from the spine) towards the end of endurance tasks indicates increased involvement of the lateral components of the erector spinae muscles, such as the longissimus. This finding can be explained by the role of the ES muscles, which are considered the main contributors to trunk extension due to their fiber orientation [30]. As the task becomes more challenging, lumbar muscle components with the highest mechanical advantage, such as the longissimus, may be preferentially recruited [14]. This can be seen as a strategy to seek for new motor solutions to preserve motor performance under the influence of fatigue. Additionally, it might also be considered as a compensatory mechanism where adjacent muscles are recruited to maintain the desired force output (e.g., hip extensor muscles).

When considering the analysis of the Activation Intensity Path, we observed that the path shifted along the *FP* direction is one order of magnitude larger than the one in the *FT* direction. This causes the global metric (Mean AIP) to be influenced mainly by the larger contribution in the *FP* direction that masks the smaller contribution given by the total path of the *FT* coordinate. When studying the behavior of the travelled path in the two single directions, we also observed that, while there is no difference in the *FP* direction between the beginning and the end, a significant increase is found in the *FT* direction towards the end of the contraction. This, again, suggests that at the beginning of the contraction there might be a more uniform spatial redistribution of the muscle activity following the length of the muscle fiber direction, but as the lumbar ES fatigues, a significant shift or adjustment is produced more laterally as a compensatory mechanism to manage fatigue.

The increase in Mean Activation Velocity, specifically in the *FT* direction, was associated with the development of muscle fatigue, indicating a quick adjustment of the recruited muscle fibers to maintain the contraction. These findings suggest that although the muscle primarily compensates for strain with slower adjustments along the *FP* direction, rapid changes in the *FT* direction serve as early signs of approaching exhaustion. Even though these lateral shifts in the *FT* direction are smaller in magnitude, they become increasingly fast as fatigue intensifies, making these velocity metrics a sensitive indicator to warn about imminent muscle exhaustion.

These results are also reflected in the three parameters (i.e., ACZ, AEZ, AMZ) that are associated with the amount of displacement of the RoA with respect to the initial centroid location. We noticed that all the variables could discriminate muscle fatigue from the beginning to the end of the contraction by showing a significant difference given by the increase in the amount of shift towards the end. Although they provide similar measures of how much the RoA shifts over time, the AMZ refers to the total area covered by the AIP, and it might indicate that there is great variability in muscle activity. On the other hand, the AEZ provides a region in which the RoA is expected to be found considering two principal directions (*FP* and *FT*). Therefore, the change in the shape of the ellipse during fatigue could offer insights into the primary directions in which muscle activity shifts. These parameters all revealed that, as fatigue occurred, the RoA displacement increased, suggesting that more regions of the lumbar ES become active with the progression of fatigue.

Initially, at the onset of the contraction, there is a uniform distribution among participants. Yet, as the lumbar ES started to fatigue, this uniformity diminished, leading to increased inter-variability among subjects. This suggests that while there may be a common adaptive strategy against fatigue, the specifics of this strategy might differ between individuals. Such variability, particularly evident when muscle fatigue intensified, might depend on several factors such as differences in muscle morphology, conditioning, or even individual tolerance to discomfort associated with fatigue.

Finally, as presented in Table II, the maximum peak value of some metrics extracted by the RoA exhibited a significant and strong correlation with endurance time. In particular, our results showed that greater spatial changes in the *FT* direction were associated with longer endurance time, and we hypothesized that the shift in this direction might be the final muscle strategy adopted immediately before exhaustion. This observation implies that the maximum values of these spatial metrics might offer valuable insights into the physiological factors affecting endurance. Such findings have the potential to provide future opportunities to predict the time to task failure. While this study offers valuable insights, it is important to acknowledge that the sample size was small and homogeneous, consisting of nine young males. Additionally, the metrics were defined and tested on isometric contraction. Although we explored varying window lengths for metric computation, future research should consider a broader range of window lengths and test these metrics also on dynamic contractions to provide a more comprehensive validation of the metrics and enhance the applicability of the findings. Finally, it will be interesting to investigate the goodness of these metrics also on other muscles during fatiguing contractions (e.g., lower, or upper limb muscles).

## V. Conclusion

By integrating parameters typically used to investigate postural control, this study introduces innovative metrics derived from HD-sEMG measurements to quantify and characterize the neuromuscular adaptations accompanying fatigue. These new metrics merge spatial and temporal information, offering new insights into muscle activation, redistribution, and adaptation during prolonged isometric contractions. The significant differences in the evolution over time of these spatial metrics, describing the RoA movement throughout muscle contraction, could be used to track the progression of muscle fatigue and potentially predict the time of exhaustion. This prediction is supported by the strong correlation between the peak values of specific metrics and endurance time. Furthermore, the observations of the lumbar erector spinae muscle revealed the importance of considering the direction of the muscle activation shift, particularly with respect to fiber orientation. This directional aspect is important because it might reflect different neuromuscular strategies: on one hand, the shift in the fiber parallel direction is needed to recruit more fibers and optimize the activation of various muscle regions to sustain the required torque level throughout the contraction; on the other hand, the movement of the RoA in the fiber transverse direction is used to extend endurance time before reaching exhaustion.

## Author Contributions

G.C.: Conceptualization, Methodology, Investigation, Data Curation, Formal Analysis, Visualization, Writing - Original Draft, Writing - Review and Editing.

M.A.: Conceptualization, Methodology, Investigation, Writing - Review and Editing.

D.F.: Conceptualization, Methodology, Resources, Validation, Writing - Review and Editing, Supervision.

S.C.: Conceptualization, Methodology, Validation, Project administration, Writing - Review and Editing, Supervision.

## Conflict of Interest

The authors declare no conflict of interest.

## Supplementary Materials

Supplementary materials
