# Supplementary material for: Novel Metrics for High-Density sEMG Analysis in the Time–Space Domain During Sustained Isometric Contractions
Source: IEEE Open J Eng Med Biol. 2024 Aug 26;5:760–8. doi: 10.1109/OJEMB.2024.3449548 (PMC11379446; doi:10.1109/OJEMB.2024.3449548)
Supplement: Supplementary materials [file supp1-3449548.pdf]

Supplementary Materials

Novel Metrics for High-Density sEMG Analysis in the Time-Space Domain during Sustained Isometric Contractions

Giovanni Corvini, Michail Arvanitidis, Deborah Falla, Silvia Conforto

These supplementary materials provide a comprehensive description of the Materials and Methods sections. It explains in detail the study design, protocol, signal processing techniques, mathematical derivation of the proposed metrics, and tests performed for the statistical analysis. The code for the computation of these metrics is also available at:
https://github.com/giovannicorvini/Novel_Metrics_for_High-Density_sEMG.git
References of the main manuscript are reported at the end of the document.

I. Materials and Methods

A. Design and Settings

This observational study consisted of three separate sessions per participant each performing different types of contraction (i.e., isometric, pseudo-dynamic and dynamic). Each session lasted approximately 2 hours and sessions were spaced at least three days apart. The analysis presented in the main manuscript focuses on data collected from a single session, specifically on the fatiguing isometric contraction. The study was conducted at the Centre of Precision Rehabilitation for Spinal Pain (CPR Spine, University of Birmingham, UK) and in accordance with the Declaration of Helsinki. The study was approved by the Ethics Committee of the University of Birmingham (approval number: ERN_2022-0514). Written informed consent was obtained from all participants before participating in the experiment. For the precise capture of physiological indicators, our study employed the High-Density Surface Electromyography (HD-sEMG), while for torque measurements an isokinetic dynamometer, provided with a specialized chair attachment, was employed. This acquisition chain was thoughtfully designed to ensure a holistic evaluation of muscle performance and fatigue. The use of High-Density sEMG contributes to providing a detailed map of muscle activation patterns by analyzing the electrical activity generated by the muscle fibers during contractions. The increased spatial resolution allows for a complete understanding of muscle behavior under stress, making it an invaluable tool for our analysis. Concurrently, the use of the Biodex system, facilitated the precise measurement of torque output, which is essential for quantifying the physical force exerted by the muscles, and consequently thereby offering a direct indicator of muscle fatigue and endurance over time.

B. Participants

Nine healthy men (mean ± SD: age 29.1 ± 3.04 years, weight 75.1 ± 18.4 kg, height 178.0 ± 9.50 cm) with no history of low back pain, hip pain, or sciatica, participated in the study. Other exclusion criteria were rheumatic or neuromuscular disorders, history of chronic respiratory or neurological problems, spinal deformity or surgery, and cardiovascular conditions.

C. Protocol

The protocol consisted of a sequence of three Maximum Voluntary Contractions (MVCs), each lasting 5 seconds, with a 1-minute rest period between them. During these three MVCs, we recorded the Maximum Voluntary Torque (MVT) from which we extracted the peak value that served to establish a baseline before initiating the fatiguing contraction. Following five minutes of rest period to ensure the participants were not fatigued, they were instructed to perform a fatiguing contraction at 60% of their MVT peak, aiming to maintain this level for as long as possible. Visual feedback, based on the exerted torque, was provided to maintain the required level of torque at the targeted level. When the exerted torque level of the participant dropped to 50% of MVT peak or less (i.e., more than 10%) for a minimum of three consecutive seconds, task failure was reached, and the experiment was stopped.

D. Surface EMG and Torque acquisition

HD-sEMG signals were detected from the right lumbar Erector Spinae (ES) with two semi-disposable adhesive grids of 64 electrodes each (OTBioelettronica, Torino, Italy, model GR08MM1305). The grids are composed of 13 rows and 5 columns of electrodes (1-mm diameter, 8-mm Inter-Electrode Distance (IED) in both directions). Notably, the upper left corner does not contain an electrode; this absence is deliberate, serving as a reference point to establish the orientation of the grid. HD-sEMG signals were amplified by a factor of 150 (Quattrocento amplifier, OTBioelettronica, #3 dB bandwidth, 0.8–500 Hz), sampled at 2048 Hz and converted to digital form by a 16-bit A/D converter with a resolution of 152 μV (input range ±5 V). Before applying the grids, an investigator identified the L5 vertebra and marked the skin 2cm lateral to this point, extending until the ~T10 vertebra following the direction of the muscle fibers. Then, the skin was prepared by shaving the area (when necessary), cleaning it with an abrasive paste (SPES Medica, Italy) to reduce skin impedance and, finally, washing it with water and drying it. The two grids were applied one immediately above the other to cover and record signals from most of the lumbar ES muscle. A reference electrode was placed on the left posterior superior iliac spine (PSIS), as shown in Figure 1. The lumbar ES was chosen because it plays an important role in lifting activities. The right side of the muscle was selected based on the dominance side of the participants, who were all right-handed. Furthermore, a study by Varrecchia et al. [25] demonstrated no significant differences in bipolar and HD-sEMG parameters between the

right and left sides of the lumbar ES during lifting tasks. This approach has also been effectively used in studies involving healthy individuals [22][31].

For the torque acquisition, an isokinetic dynamometer with the chair attachment (Biodex Corporation, Biodex System 3 Pro model, USA) was used. The torque signal was acquired during the back extension movements through a BNC cable attached to one of the auxiliary inputs of the sEMG amplifier. For this reason, being directly acquired by the sEMG acquisition software, the sampling frequency of the torque was equal to that of the sEMG signals, that is 2048 Hz. Before the beginning of each experiment, this device was calibrated. The torque signal was used to provide the participant with visual feedback of the exerted torque. The visual feedback, displayed on a screen placed in front of the participants (approximately 1.5 meters), showed the exerted torque, represented by a dynamic blue bar (increasing or decreasing according to the participant's muscle output), and the reference torque, represented by a constant red line at 60% of MVC. The participant should exert torque until the blue bar reached the red line and keep it constant as much as possible.

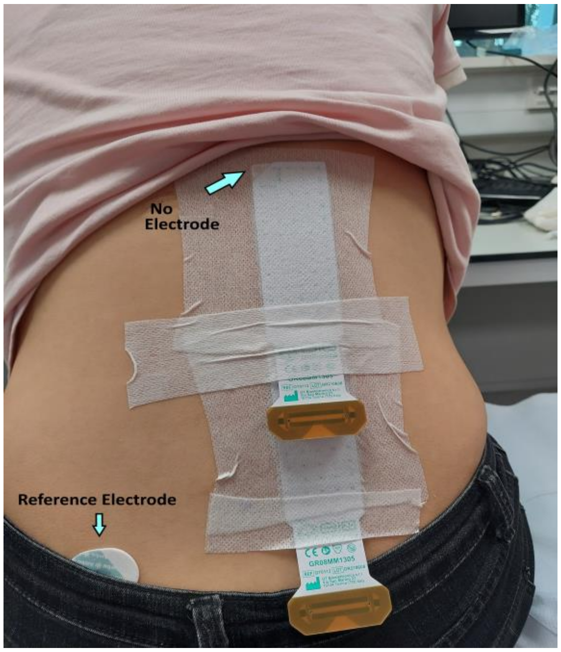

Figure 1 HD-sEMG grids applied on the right part of the lumbar Erector Spinae and the reference electrode.

E. Data Processing

To calculate the Region of Activation (RoA) as the weighted centroid of the electrical activity greater than 70% of the local maximum, we follow this approach. First, we denoted $A(x,y,n)$ as the electrical activity at a point (x, y) at time n, where x and y are the spatial coordinates and n indicates the time instant. We refer to x and y as Fiber Transverse (*FT*) and Fiber Parallel (*FP*), respectively. At each time instant n, we found the local maximum activity level $A_{max}(n)$, and we defined the active muscle Region $R(x,y,n)$ at time n as the set of points where the electrical activity is greater than 70% of the local maximum:

$$R(x,y,n) = A(x,y,n) > 0.7 * A_{max}(n) \qquad (1)$$

All the points falling below this threshold $(0.7 * A_{max}(t))$ were set to zero. To calculate the weighted centroid along the two directions (*FT* and *FP*), we used the following formulas:

$$\mathrm{RoA^0}_{FT}(n) = \frac{\sum_{y=1}^{Y}\sum_{x=1}^{X} x * R(x,y,n)}{\sum_{X,Y} R(x,y,n)} \qquad (2)$$

$$\mathrm{RoA^0}_{FP}(n) = \frac{\sum_{y=1}^{Y}\sum_{x=1}^{X} y * R(x,y,n)}{\sum_{X,Y} R(x,y,n)} \qquad (3)$$

where X and Y are the total number of columns (x) and rows (y) of the HD-sEMG grid, respectively.

The x- and y-coordinates of the original time series (RoA^0_{FT}, RoA^0_{FP}) were smoothed with a low pass filter at 10Hz (3rd order Butter) before deriving the spatial metrics. For the calculation of the metrics using the entire length of the signal, we used the discretized version of the RoA coordinates, referring to the time instant *n* (also called time point). From the entire length of the signal, we extracted three 10-second segments (S) at the beginning (T 1), the middle (T 2) and the end (T 3) of the contraction for the statistical analysis. In the metric definitions we will use *K* to refer to the total number of points within one of these segments S, to differentiate it from the total number of points *N* corresponding to the original length of the signal.

F. Metric Definitions

Using the formula from Prieto et al. [20], we derived and adapted several parameters typically calculated from the Centre of Pressure (CoP) to study postural control. In the following, we derived all the metrics from the RoA coordinates (RoA^0_{FT}, RoA^0_{FP}), but the same definitions would apply to the coordinates of the Center of Gravity (CoG), defined as the weighted centroid of the total electrical activity.

First, we define the Activation Intensity Distance (AID), a vector representing the distance of each point of the muscle's Region of Activation along both directions (RoA_{FP}, RoA_{FT}) from a point representing the initial muscle activity. This initial centroid can be determined either at rest or at the beginning of a muscle contraction, depending on the objective of the study. In our case we aimed to assess changes relative to the beginning of muscle activity, therefore we arbitrarily chose to use as an initial centroid the CoG averaged in the first 20% of the duration of the contraction (that is different for each subject according to the total endurance time). This AID distance vector is computed for the entire length of the contraction at each time point *n*, and it is mathematically expressed as:

$$\mathrm{AID}[n] = \sqrt{\mathrm{RoA}_{FT}[n]^2 + \mathrm{RoA}_{FP}[n]^2} \quad \textit{with } n=1,\ldots,N \qquad (4)$$

where RoA_{FT} and RoA_{FP} are the demeaned coordinates of the RoA centroid, and *N* is the total number of points of the original signal length.

These two coordinates are calculated according to the following equations:

$$\text{RoA}_{FT}[n] = \text{RoA}^0_{FT}[\text{n}] - \overline{\text{CoG}_{FT}} \tag{5}$$

$$\text{RoA}_{FP}[n] = \text{RoA}^0_{FP}[\text{n}] - \overline{\text{CoG}_{FP}} \tag{6}$$

where RoA^0_{FT} is the original x-coordinate of the RoA (filtered) and $\overline{\text{CoG}_{FT}}$ is the mean of the x-coordinate extracted by the Centre of Gravity in the first 20% of the contraction. The same holds for the RoA^0_{FP} and $\overline{\text{CoG}_{FP}}$ being the y-coordinate.

Then, we define the Mean Activation Intensity Distance (Mean AID), which is the average distance of the RoA displacement from the initial centroid in the three selected time segments S. The Mean AID is computed according to:

$$\text{Mean AID} = \frac{1}{K}\sum_{k=1}^{K} \text{AID}[k] \tag{7}$$

where K is the total number of points contained in a specific time segment S (in this paper S=3, each of 10 seconds). The AID metric could also be computed along each single direction (Transverse or Parallel) as:

$$\text{Mean AID}_{FT} = \frac{1}{K}\sum_{k=1}^{K} |\text{RoA}_{FT}[k]| \tag{8}$$

where AID_{FT} is the mean absolute value of the *FT* time series calculated in a selected time interval consisting of K points, representing the average *FT* Activation Intensity Distance (AID) from the initial centroid (i.e., the mean CoG_{FT} in the first 20% of the contraction). The same applies to the Fiber-Parallel component.

The Total Activation Intensity Path (AIP) represents the entire path travelled by the RoA along both directions and it is defined as follows:

$$\text{Total AIP} = \sum_{n=1}^{N-1} \sqrt{\begin{matrix}(\text{RoA}^0_{FT}[n+1] - \text{RoA}^0_{FT}[n])^2 + \\ (\text{RoA}^0_{FP}[n+1] - \text{RoA}^0_{FP}[n])^2\end{matrix}} \tag{9}$$

This global measure can be calculated singularly for the *FT* coordinate (as well as for the *FP* coordinate by substituting the *FT* with *FP*). The total AIP_{FT} (or AIP_{FP}) indicate the total length of the distance travelled by the RoA along the *FT* (or *FP*) direction and it can be approximated by the sum of the distances between consecutive points extracted from the *FT* (or *FP*) temporal series, according to the formula:

$$\text{Total AIP}_{FT} = \sum_{n=1}^{N-1} \left| \widehat{\text{RoA}_{FT}}^0[n+1] - \widehat{\text{RoA}_{FP}}^0[n] \right| \tag{10}$$

where the two original RoA coordinates $(\widehat{\text{RoA}_{FT}}^0, \widehat{\text{RoA}_{FP}}^0)$ were adjusted by removing the trend of the time series within the specific time window considered. For the definition of this metric, it was essential to use the original time series to avoid any potential biases that might arise from mean removal during calculations. However, the trend of the time series, as determined within each respective time window where the parameters were computed, was subtracted. This adjustment ensured that any differences between the RoA coordinates due to the magnitude did not influence the observed shifts in both directions, thereby preserving the integrity of the parameter calculations.

Using the Total AIP, it is possible to define and calculate the Mean Activity Intensity Velocity (AIV), which represents the average speed of the RoA over a specific time interval composed of K points. It is defined as:

$$\text{Mean AIV} = \frac{\text{Total AIP}}{K} \tag{11}$$

where K is the total number of points in the considered time interval for the parameter calculation. The AIP metric can also be computed on a single direction as follow:

$$\text{Mean AIV}_{FT} = \frac{\text{Total AIP}_{FT}}{K} \tag{12}$$

The Mean AIV_{FT} represents the average velocity of the *FT* components of the RoA in a specific time interval composed of K points extracted from the total length of the time series. The same applies for the *FP* component.

Then we defined two metrics able to identify the total space covered by the travelling RoA coordinates.

Initially, we introduced the concept of Zone, an area enclosing most of the points of the AIP. We define the Activation Ellipse Zone (AEZ), which represents the area of the 95% bivariate confidence ellipse that shall enclose approximately 95% of the points of the RoA AIP. For this definition, the assumption is that that the number of data points used is large enough such that the ratio $\frac{n-1}{n-2} \approx 1$. The formula for AEZ is given by:

$$\text{AEZ} = 2\pi F_{0.05[2,n-2]} \sqrt{[std^2_{FT} std^2_{FP} - sd^2_{FTFP}]} \tag{13}$$

In this equation, $F_{0.05[2,\, q-2]}$ represents the F-statistic at the 95% confidence level for bivariate distribution with n points, and for large sample sizes (n > *120*) this value is set to 3.00 [20]. The std_{FT} and std_{FP} are the standard deviations of the *FT* and *FP* time series, respectively, while std_{FTFP} is the covariance. By using the equation 5 and 6, these variables can be computed as:

$$std_{FT} = \sqrt{\frac{1}{K-1}\sum_{k=1}^{K} RoA_{FT}[k]^2} \quad (14)$$

$$std_{FTFP} = \frac{1}{K}\sum_{k=1}^{K} RoA_{FT}[k] RoA_{FP}[k] \quad (15)$$

where std_{FP} can be computed by substituting in equation 14 the RoA_{FT} with RoA_{FP}.

Similarly, we also define the Activation Circle Zone (ACZ). This zone denotes the projected area of a circle whose radius is the 95% percentile of the AID; in simpler terms, this circle includes approximately 95% of the points of the RoA AIP. For this metric the assumption is that all Activation Intensity Distances follow a normal distribution. The formula to compute the ACZ is:

$$\text{ACZ} = \pi(\text{Mean AID} + z_{0.05}\, s_{AID})^2 \quad (16)$$

In this equation the $z_{0.05}$ refers to the z-score associated with a significance level of $\alpha = 0.05$ in a standard normal distribution and it is equal to 1.645. The standard deviation of the AID can be computed as:

$$std_{AID} = \sqrt{\frac{1}{K}\sum_{k=1}^{K} \text{AID}^2[k] - \text{Mean AID}^2} \quad (17)$$

Finally, we define the Activation Movement Zone (AMZ), which estimates the total region covered by the RoA AIP per unit of time. This metric is computed by summing the area of the triangles formed by two consecutive points on the AIP and the initial centroid. It can be thought of as being proportional to the combination of Mean AID and Mean AIV. The formula to compute the AMZ is:

$$\text{AMZ} = \frac{1}{2K}\sum_{k=1}^{K-1} \left| \begin{matrix} (\widehat{\text{RoA}_{FT}{}^{0}[k}+1] - \widehat{\text{RoA}_{FP}{}^{0}}[k] - \\ (\widehat{\text{RoA}_{FT}{}^{0}}[k] - \widehat{\text{RoA}_{FP}{}^{0}[k}+1] \end{matrix} \right| \quad (18)$$

where K is the total number of points k in the selected time interval in which the metric is computed and, as for the Total AIP, we use the original RoA coordinates without the trend in the specific time interval K.
Finally, by using the standard deviations along the two directions *FT* and *FP*, we define the Activation Intensity Spread (*AIS*) as:

$$\text{AIS} = \sqrt{[std_{FT}^2 + std_{FP}^2]} \quad (19)$$

This metric provides information on the amount of the RoA fluctuations around its initial position over time. A higher value indicates more variability in the muscle activation patterns. All these metrics have been normalized to the height of the subject.

These metrics could be grouped according to the information they provide. Specifically, we have AID that represents the distance reached from the RoA within the HD-sEMG grid. It indicates the different points in the grid where Motor Units have been recruited relative to the center of activation at the beginning of the contraction.

Then we have the AIP and AIV, which are directly proportional, that express the cumulative distance travelled by the RoA and its velocity. These metrics do not provide information on the specific location reached but quantify the extent of fiber recruitment across different muscle regions. The AIV might provide indirect information on the rate of the selective fibers recruitment in different muscle regions. For example, if the entire muscle was completely active throughout the contraction, these values would be low, indicating more uniform activation of the muscle below the grid and suggesting a more static distribution of recruited motor units.

Finally, we have metrics related to the Zones in which they are active, and specifically ACZ, AEZ, AMZ and AIS. The first two metrics provide a global view of the RoA movements within the HD-sEMG grid. ACZ captures the overall shift in all directions, while AEZ highlights movement in specific directions, providing insight into the directional nature of muscle activation.

AMZ instead provides information on the oscillation of the RoA with respect to an initial point, and AIS quantifies the variability and spread of activation intensity. The latter, for example, could reveal asymmetries in muscle activation patterns that might be related to specific functional or pathological conditions. Together, these metrics might offer a robust toolset for analyzing muscle function, coordination, control, and adaptation in both research and clinical settings.

In addition to the calculation of these metrics, we also computed the parameters commonly analyzed in previous studies, which are the averaged value of the RoA coordinates in the specific time intervals, and the RoA shift calculated as the modulus of the RoA displacement in *FT* and *FP* directions at the beginning (1% endurance time) and at the end (99% endurance time) of the contraction [17]. Additionally, we also determined the amount of the exerted torque (normalized with respect to the peak value extracted from the MVT), the Coefficient of Variation of the torque (CoV of torque), the envelope, (mean value across all channels of the grid) and the AIF (mean value across all channels of the grid).

Given the variability of the contraction duration among individuals, we will refer to different phases of the contraction in terms of their relative time percentages. For clarity, 0% and 100% correspond to the two “quasi-zero activity” window, 1% of the contraction time refers to its beginning, while 99% denotes its conclusion. In this study, based on the minimum total duration (around 70 seconds), we arbitrarily selected three 10-second intervals to capture the essential phases of the contraction. From these windows (i.e., initial 10 seconds (1%), middle 10 seconds (50%) and the last 10 seconds (99%), we extracted all the described metrics and performed the statistical analysis.

REFERENCES

[1] R. M. Enoka and J. Duchateau, "Muscle fatigue: what, why and how it influences muscle function: Muscle fatigue," The Journal of Physiology, vol. 586, no. 1, pp. 11–23, Jan. 2008, doi: 10.1113/jphysiol.2007.139477.

[2] Wan, J., Qin, Z., Wang, P., Sun, Y., & Liu, X. (2017). Muscle fatigue: General understanding and treatment. Experimental & Molecular Medicine, 49(10), e384–e384. https://doi.org/10.1038/emm.2017.194

[3] C. J. De Luca, "Myoelectrical manifestations of localized muscular fatigue in humans," Crit Rev Biomed Eng, vol. 11, no. 4, pp. 251–279, 1984.

[4] M. González-Izal et al., "Electromyographic models to assess muscle fatigue," Journal of Electromyography and Kinesiology, vol. 22, no. 4, pp. 501–512, Aug. 2012, doi: 10.1016/j.jelekin.2012.02.019.

[5] M. Cifrek et al., "Surface EMG based muscle fatigue evaluation in biomechanics," Clinical Biomechanics, vol. 24, no. 4, pp. 327–340, May 2009, doi: 10.1016/j.clinbiomech.2009.01.010.

[6] A. Luttmann, M. Jager, and Laurig, Wolfgang, "Electromyographical indication of muscular fatigue in occupational field studies," International Journal of Industrial Ergonomics, 2000.

[7] A. Gallina, R. Merletti, and M. Gazzoni, "Uneven spatial distribution of surface EMG: what does it mean?" Eur J Appl Physiol, vol. 113, no. 4, pp. 887–894, Apr. 2013, doi: 10.1007/s00421-012-2498-2.

[8] M. J. Zwarts and D. F. Stegeman, "Multichannel surface EMG: Basic aspects and clinical utility," Muscle Nerve, vol. 28, no. 1, pp. 1–17, Jul. 2003, doi: 10.1002/mus.10358.

[9] T. M. M. Vieira, R. Merletti, and L. Mesin, "Automatic segmentation of surface EMG images: Improving the estimation of neuromuscular activity," Journal of Biomechanics, vol. 43, no. 11, pp. 2149–2158, Aug. 2010, doi: 10.1016/j.jbiomech.2010.03.049.

[10] Abboud, J., Ducas, J., Marineau-Bélanger, É., & Gallina, A. (2023). Lumbar muscle adaptations to external perturbations are modulated by trunk posture. European Journal of Applied Physiology, 123(10), 2191–2202. https://doi.org/10.1007/s00421-023-05223-2

[11] K. Tucker et al., "Electromyographic mapping of the erector spinae muscle with varying load and during sustained contraction," Journal of Electromyography and Kinesiology, vol. 19, no. 3, pp. 373–379, Jun. 2009, doi: 10.1016/j.jelekin.2007.10.003.

[12] Bigland-Ritchie, B., Rice, C. L., Garland, S. J., & Walsh, M. L. (1995). Task-Dependent Factors in Fatigue of Human Voluntary Contractions. In S. C. Gandevia, R. M. Enoka, A. J. McComas, D. G. Stuart, C. K. Thomas, & P. A. Pierce (Eds.), Fatigue: Neural and Muscular Mechanisms (pp. 361–380). Springer US. https://doi.org/10.1007/978-1-4899-1016-5_29

[13] Champagne, A., Descarreaux, M., & Lafond, D. (2008). Back and hip extensor muscles fatigue in healthy subjects: Task-dependency effect of two variants of the Sorensen test. European Spine Journal, 17(12), 1721–1726. https://doi.org/10.1007/s00586-008-0782-y

[14] Marineau-Bélanger, E., Vaurs, M., Roy, J., O'Shaughnessy, J., Descarreaux, M., & Abboud, J. (2023). Fatigue task-dependent effect on spatial distribution of lumbar muscles activity. Journal of Electromyography and Kinesiology, 73, 102837. https://doi.org/10.1016/j.jelekin.2023.102837

[15] Abboud, J., Nougarou, F., Loranger, M., & Descarreaux, M. (2015). Test-Retest Reliability of Trunk Motor Variability Measured By Large-Array Surface Electromyography. Journal of Manipulative and Physiological Therapeutics, 38(6), 359–364. https://doi.org/10.1016/j.jmpt.2015.06.007

[16] D. Falla and A. Gallina, "New insights into pain-related changes in muscle activation revealed by high-density surface electromyography," Journal of Electromyography and Kinesiology, vol. 52, p. 102422, Jun. 2020, doi: 10.1016/j.jelekin.2020.102422.

[17] D. Farina et al., "The change in spatial distribution of upper trapezius muscle activity is correlated to contraction duration," Journal of Electromyography and Kinesiology, vol. 18, no. 1, pp. 16–25, Feb. 2008, doi: 10.1016/j.jelekin.2006.08.005.

[18] K. Tucker et al., "Electromyographic mapping of the erector spinae muscle with varying load and during sustained contraction," Journal of Electromyography and Kinesiology, vol. 19, no. 3, pp. 373–379, Jun. 2009, doi: 10.1016/j.jelekin.2007.10.003.

[19] A. Sanderson et al., "Variation in the spatial distribution of erector spinae activity during a lumbar endurance task in people with low back pain," J. Anat., vol. 234, no. 4, pp. 532–542, Apr. 2019, doi: 10.1111/joa.12935.

[20] T. E. Prieto et al., "Measures of postural steadiness: differences between healthy young and elderly adults," IEEE Trans. Biomed. Eng., vol. 43, no. 9, pp. 956–966, Sep. 1996, doi: 10.1109/10.532130.

[21] F. Quijoux et al., "A review of center of pressure (COP) variables to quantify standing balance in elderly people: Algorithms and open-access code*," Physiological Reports, vol. 9, no. 22, Nov. 2021, doi: 10.14814/phy2.15067.

[22] Arvanitidis, M., Jiménez-Grande, D., Haouidji-Javaux, N., Falla, D., & Martinez-Valdes, E. (2022). People with chronic low back pain display spatial alterations in high-density surface EMG-torque oscillations. Scientific Reports, 12(1), 15178. https://doi.org/10.1038/s41598-022-19516-7

[23] Georgakis, A., Stergioulas, L. K., & Giakas, G. (2003). Fatigue analysis of the surface EMG signal in isometric constant force contractions using the averaged instantaneous frequency. IEEE Trans. on Biomed. Engin, 50(2), 262-265. https://doi.org/10.1109/TBME.2002.807641

[24] G. Corvini, A. Holobar, and J. C. Moreno, "On repeatability of MU fatiguing in low-level sustained isometric contractions of tibialis anterior muscle," in *Proc. Int. Conf. NeuroRehabilitation*, 2022, vol. 28, pp. 909–913, doi: 10.1007/978-3-030-70316-5_145. 631

[25] Varrecchia, T., Ranavolo, A., Conforto, S., De Nunzio, A. M., Arvanitidis, M., Draicchio, F., & Falla, D. (2021). Bipolar versus high-density surface electromyography for evaluating risk in fatiguing frequency-dependent lifting activities. Applied Ergonomics, 95, 103456. https://doi.org/10.1016/j.apergo.2021.103456

[26] Martinez-Valdes, E., Wilson, F., Fleming, N., McDonnell, S.-J., Horgan, A., & Falla, D. (2019). Rowers with a recent history of low back pain engage different regions of the lumbar erector spinae during rowing. Journal of Science and Medicine in Sport, 22(11), 1206–1212. https://doi.org/10.1016/j.jsams.2019.07.007

[27] Falla, D., Gizzi, L., Tschapek, M., Erlenwein, J., & Petzke, F. (2014). Reduced task-induced variations in the distribution of activity across back muscle regions in individuals with low back pain. Pain, 155(5), 944–953. https://doi.org/10.1016/j.pain.2014.01.027

[28] M. Arvanitidis et al., "Spatial distribution of lumbar erector spinae muscle activity in individuals with and without chronic low back pain during a dynamic isokinetic fatiguing task," Clinical Biomechanics, vol. 81, p. 105214, Jan. 2021, doi: 10.1016/j.clinbiomech.2020.105214.

[29] Abboud, J., Kuo, C., Descarreaux, M., & Blouin, J.-S. (2020). Regional activation in the human longissimus thoracis pars lumborum muscle. The Journal of Physiology, 598(2), 347–359. https://doi.org/10.1113/JP278260

[30] McGill, S. M., Santaguida, L., & Stevens, J. (1993). Measurement of the trunk musculature from T5 to L5 using MRI scans of 15 young males corrected for muscle fibre orientation. Clinical Biomechanics, 8(4), 171–178. https://doi.org/10.1016/0268-0033(93)90011-6

[31] Arvanitidis, M., Jiménez-Grande, D., Haouidji-Javaux, N., Falla, D., & Martinez-Valdes, E. (2024). Low Back Pain–Induced Dynamic Trunk Muscle Control Impairments Are Associated with Altered Spatial EMG–Torque Relationships. Medicine & Science in Sports & Exercise, 56(2), 193–208. https://doi.org/10.1249/MSS.0000000000003314
